# Storied reflections: Development of a longitudinal interdisciplinary curriculum to improve patient-provider communication

**DOI:** 10.1016/j.pecinn.2023.100170

**Published:** 2023-05-26

**Authors:** Nicole Defenbaugh, Lorraine A. Dickey, Vivian C. Foulke, James P. Orlando

**Affiliations:** a University of Health Sciences and Pharmacy, 1 Pharmacy Place, St. Louis, Missouri 63110, United States of America; b St Luke's University Health Network, 801 Ostrum Street, Bethlehem, PA 18015, United States of America; c Lehigh Valley Hospital, 1200 South Cedar Crest Blvd, Allentown, PA 18103, United States of America; d Independent Scholar

**Keywords:** Interdisciplinary, Narrative, Medical education, Patient-healthcare provider communication, Listening, Relationship-centered care, Resiliency, Wellness

## Abstract

**Objective:**

This article details the development of an interdisciplinary graduate medical education (GME) narrative curriculum.

**Methods:**

Descriptive statistics were conducted for the narrative session surveys. Two separate qualitative analyses were conducted. First, content and thematic analyses of the open-ended questions in the survey using NVIVO software occurred. Second, an inductive analysis of the participants' 54 stories was performed to identify unique themes not related to the prompt topics.

**Results:**

Quantitative survey results demonstrated that 84% of learners' felt the session benefited their personal or professional sense of wellbeing and resilience, 90% of learners believed the sessions aided in their ability to listen more effectively, and 86% of learners could apply what they practiced or witnessed. Qualitative analysis of survey data showed learners focused on patient care and listening. Thematic analysis of participants' narratives revealed strong feelings and emotions, struggles with time management, increase in self- and other-awareness, and challenges managing job-life balance.

**Conclusion:**

The longitudinal interdisciplinary Write-Read-Reflect narrative exchange curriculum is cost-effective, sustainable, and demonstrably valuable to learners and their program directors across multiple disciplines.

**Innovation:**

The program was designed for 4 graduate programs' learners to simultaneously experience a narrative exchange model to improve patient-provider communication, support professional resilience, and deepen relationship-centered care skills.

## Highlights

• A longitudinal, interdisciplinary narrative program was developed for medical education.
• Learners gained insight regarding the importance of teamwork and patient-healthcare provider communication.
• Learners were able to listen more effectively to others.
• Learners developed a sense of wellbeing and resilience.
• Learners could apply what they practiced during narrative sessions to their clinical encounters.

## 1 Introduction

“She is breathing fine on room air,” says ENT.

As I interview her [patient] with a virtual Ukrainian interpreter,

she grimaces every time she squeaks out a word.

Between agonizing answers to my banal questions,

I hear every breath whistle through her drinking straw-sized remnants of a trachea.

“I'm worried about her airway” [I say].

“Just relax. We'll get the biopsy and take it from there”. [ENT specialist].

*--- Narrative session learner*

Interdisciplinary teamwork continues to garner attention in medical education [[1], [2], [3], [4], [5], [6], [7]]. Medical educators recognize the importance of their learners knowing how to work in teams to improve patient safety and clinical care. But not all learners acquire medical knowledge the same way because of differences in medical techniques and cultures that occur across disciplines [5]. The opening story demonstrates how the learner coordinates with a different department (i.e., ENT) to provide effective patient care. The learner shares their worry about the patient and is reassured by the ENT specialist who states, “just relax” followed by an explanation of the care they will provide. The story, shared among other learners from different disciplines during a narrative session, reveals the storyteller's awareness of interdisciplinary teamwork.

Interdisciplinary teamwork and collaborative learning in undergraduate medical education (UME) is often conducted by grouping learners' interests in different specialties or disciplines [5,8]. In this way, learners gain knowledge and insight about interdisciplinary teamwork through reflective thinking [8]. Reflection has been found to play a critical role in learning [9] and can also assist learners in developing a myriad of skills in critical areas such as emotional intelligence and communication [10]. Reflective practice at the graduate medical education (GME) level is equally important. In GME, learning about interdisciplinary teamwork frequently occurs during clinical rotations. For example, interdisciplinary learning occurs at bedside rounds [6], conference calls [1] or through the development of simulation sessions [7] and certificate programs [4]. Cross-disciplinary education at the graduate level might occur in post-clinical education sessions using faculty from different programs and reflective activities [7]. Cross-learner-level learning and longitudinal education programs can result in great insights regarding interdisciplinary teamwork. However, this type of learning often occurs in short, controlled education settings such as one-day workshops [2]. Longitudinal education with learners from different disciplines *and* learning levels has profound advantages, especially if it is learner-driven education [6]. However, this type of education faces unique challenges (e.g., scheduling) [3] for cross-disciplinary learners to be present at the same time. Learning about interdisciplinary teamwork from other residents using reflective activities can be educationally beneficial but is difficult to coordinate and therefore rare. Our innovative program aims to fill the gap by providing a longitudinal, interdisciplinary and reflective program in medical education.

Narrative medicine is an important discipline in medical education [11] and a useful methodology for studying learner identity formation [12,13]. It is also a vital teaching tool for learner self-reflection [[14], [15], [16], [17], [18], [19]] and skill development in areas such as patient-provider communication and teamwork [15], mindfulness and reflection [20,21], relationship-centered care [22], empathy and burnout [17], cultural competence [23], professionalism [24], and interprofessionalism [25,26], topics in social and behavioral sciences [27], and interdisciplinary collaboration [8,28]. Because of the two lead authors' background using narrative methods of engagement in healthcare, we chose to use the Write-Read-Reflect (WRR) narrative tool for this project. The WRR tool was first published in 2011 [29] and later used in an online format at the start of the COVID-19 pandemic [30]. The WRR tool helps facilitate personal stories from healthcare professionals to transform the patient experience and teaches participants foundational skills in patient-centered care such as empathic listening and reflection. The WRR process also allows for decompression and enhances resilience by addressing challenges to teamwork and professionalism [29,30].

In the 2019–2020 academic year, an IRB-approved pilot narrative program was developed with pastoral care (PC) and hospital and palliative medicine (HPM). The program emerged because of the two program directors' mutual interest in using reflective practice to teach about common curriculum issues such as empathy, interdisciplinary teamwork, end of life care, and imminently dying patients. This interdisciplinary narrative approach focused on the learners' stories to explore common clinical challenges and understand the importance of effective patient-provider communication (PPC). “Patient-provider communication is the shared and mutually understood exchange of information between a patient and their provider” and teaches skills such as shared respect, patient autonomy and meaningful interaction [37]. Learners found their experience with the narrative curriculum consequential to their medical education based on their response to the use of two, validated mixed-methods surveys [29]. Based on this favorable feedback, data was collected again in the 2020–21 academic year and a third GME program director, from psychiatry, joined the project. Unfortunately, in this academic year the pandemic prevented a full set of data collection. However, preliminary results were submitted to The Center to Advance Palliative Care (CAPC) for the 2021 John A. Hartford Foundation Tipping Point Challenge and was recognized with a Silver Award. In the 2021–2022 academic year, a fourth program, family medicine, joined the project.

This article details the narrative project and its related research. This longitudinal, interdisciplinary reflective curriculum innovation was designed for learners from four programs to simultaneously experience a narrative exchange model designed to improve patient- provider communication, support professional wellness and resilience, and deepen relationship-centered skills.

## 2 Method

### 2.1 Setting and participants

A one, 2-h block session each month was agreed upon by the program directors (PDs) from all programs. PDs remained flexible with the timing of their educational curriculum to ensure learners could attend the scheduled sessions. The narrative sessions were built into each programs' curriculum so learners had dedicated time in their schedules to attend the narrative sessions. And, the PDs gave learners the option of opting out and engaging in alternative learning activities if they chose not to participate in the narrative program. For the first session of the year, we conducted two prompts. The first prompt was a “name” prompt asking participants to share a story related to their name. The prompt stated, “Take 3 minutes to write about any part of your name (e.g., last name, first name) that you felt was particularly difficult/challenging or alternatively uplifting/inspiring”. The participants then took turns reading their story verbatim to each other. It helped the participants get to know each other in a relatively quick process. We then moved on to the scheduled prompt on compassion.

The narrative project curriculum consisted of 7 scheduled, monthly, in-person 90-min narrative sessions from September 2021 to April 2022. A total of 23 learners started the program: 3 fellows from HPM (4 started, 1 subsequently left the program), 4 residents from pastoral care, 6 PGY-2 residents from psychiatry, and 9 PGY-2 residents from two different family medicine programs. For each session, the larger group was randomly divided into smaller groups of 4–8 participants with at least one learner from each program in every small group. This exempt study was approved by the Institutional Review Board (IRB).

### 2.2 Phases of intervention

The narrative project consisted of three phases: selection of project topics and development of prompts, running the narrative sessions, assessing the sessions. Topics were chosen based on a review of the programs' curricula, and prompts for each session were based on these common topics. Prompts started with less emotionally charged topics and progressed to more difficult, thought-provoking topics. The academic year for the narrative program was September to April. The prompt topics were compassion, stressors and supports, interdisciplinary team dynamics, diversity (e.g., bias, judgement), working with the imminently dying, ethics and moral distress, and religion and spirituality. PDs were invited to review the list of prompts to suggest changes, additions, or deletions prior to the start of the academic year. Unfortunately the session on diversity occured on a "snow day" (inclement weather). No data was collected.

Participants responded to a different prompt topic for each session (see Table 1). Though the topics varied from session to session, the format of the prompt always remained the same [30] allowing participants to answer from either a challenging/difficult or uplifting/inspiring perspective. One of the facilitators would read the prompt out loud, pause, and then read it again. An example of a prompt is as follows: “Take 3 minutes to write about an experience you've had with compassion that you felt was either difficult/challenging *or alternatively* uplifting/inspiring”.

**Table 1:** Session Prompt Topics.

Session Prompt Topics
Compassion
Stressor and supports
Interdisciplinary team dynamics
Imminently dying
Ethics and moral distress
Religion and spirituality

Facilitators routinely set the safety and expectations of confidentiality for every session.

Learners were invited to write for 3 min on the prompt and were then asked to read their story verbatim to the small group. After reading, facilitators asked the following questions of the author: *How did it feel to write that story? How did it feel to hear your words?* The facilitator then asked the following questions of the listeners: *How did it feel to listen to that story? What about that story makes you want this person on your team?*

### 2.3 Outcomes measured

Quantitatively, a previously published survey was used to measure learners' responses to 3 statements. The statements were meant to understand if learners felt their narrative experience resulted in clinical learnings and insights. The session survey and end of project survey were based on previously published work [29] and slightly adapted for these participants.

Qualitatively, we developed three open-ended questions on both surveys: 1. Please take a moment to list one or two learnings you will take away today, 2. How will you apply what you learned here today within your role in your program or personal life? and 3. Do you have any other comments about today's narrative experience? We emphasized the value of their stories and encouraged them to write freely. Learners were invited to leave their narratives at the end of each session. We recognized some learners wanted to take the story with them and not leave it. Therefore, the number of narratives collected does not reflect the number of participants at each session.

### 2.4 Analysis of outcomes

Descriptive statistics were conducted for the narrative project session survey. This survey was used at the end of each session and conducted for the end of project survey. Two separate qualitative analyses were also conducted. First, authors 1 and 2 engaged in thematic analysis [31] of the open-ended questions in the survey using NVivo software version 1.6.2 (QSR International). A content analysis was conducted to identify common words and phrases used in learners' response to the open-ended survey questions. After the initial analysis, a codebook was developed. The codebook was then used to code the survey responses into common themes.

## 3 Results

### 3.1 Survey results

A total of 70 GME learner interactions occurred during the six, in-person narrative sessions. Of these 70 learner interactions, 69 surveys were returned for a return rate of 98.5%. After each narrative session, participants were asked to complete a post-session survey and respond to three statements about the impact of the session on their wellbeing and resilience, ability to listen, and application of what they learned to their personal or professional life. Questions used a Likert scale from 1 to 5 (1 = strongly disagree, 5 = strongly agree). However, surveys were not collected for the last session on religion and spirituality. There was not enough time alloted after the final session for participants to complete both the end of project survey and post-session survey. The results from five sessions and the end of project survey are shown in Table 2. Results show that 84% of participants (58 of 69 surveys) “probably agree” or “strongly agree” that their narrative experience after each session benefited their personal or professional sense of wellbeing and resilience. In response to the question about how the narrative session benefited their ability to listen more effectively, 90% of participants (62 of 69 surveys) stated “probably agree” or “strongly agree”. Finally, results revealed that 86% of participants (59 of 69 surveys) “probably agree” or “strongly agree” that they left with the ability to apply what they practiced or witnessed during the narrative session to their personal or professional life.

**Table 2:** Post-Session and End of Project Survey Results.

Impact statement survey results by month	Family Medicine (FM)	Hospice & Palliative Medicine (HPM)	Pastoral Care (PC)	Psychiatry (PSY)
Wellbeing Statement				
Sept	5	4	4	4
Oct	5	5	5	5
Nov	5	5	4	N/A
Feb	5	4	4	5
Mar	N/A	N/A	5	5
April (End of project survey)	4	1	3	4
	FM	HPM	PC	PSY

Pistening Statement				
Sept	4	4	4	3
Oct	4	5	5	5
Nov	5	5	4	N/A
Feb	5	4	4	4
Mar	N/A	N/A	5	5
April (End of project survey)	3	2	3	4
	FM	HPM	PC	PSY

Application Statement				
Sept	4	3	4	5
Oct	4	5	5	5
Nov	5	5	4	N/A
Feb	5	4	4	5
Mar	N/A	N/A	5	5
April (End of project survey)	3	2	4	4

An end of project survey was given to participants. A total of 13 GME learners participated and all attendees returned the survey. It is important to note that one learner left their program midway through the academic year and did not return. We used their data up to the time of withdraw because their stories remain valid as a learner of the program and participant in the study. The final survey had the same three questions as the individual session surveys with slightly different wording to reflect the learners' participation in the entire project. The results represent a return rate of 59% (13 of 22) of total project participants and that 54% of learners “probably agree” or “strongly agree” to all three questions regarding their overall narrative experience. Because the final session also included an end of program celebration, we received fewer end-of project surveys compared to the other sessions.

In addition to the three Likert-scale questions, participants were also asked two open-ended questions. The first question asked participants for one or two learnings they will take away from the overall experience. The second question asked how they will apply what they learned. Using NVivo, the authors developed a heat map highlighting the number of responses coded to different themes. The major themes that emerged from the first question related to learnings were listening (19%), wellness (15%) and perspective (15%). The major themes from the second question revealed that participants were going to apply what they learned to patients (35%), listening (12%) and interdisciplinary teamwork (12%) with additional themes including writing, use of words, and perspective.

### 3.2 Analysis of learner narratives

The richest data came from the participants' personal stories that were handwritten and shared during the narrative sessions. In addition to the prompt themes developed for each session, we wanted to further understand what the participants' narratives revealed about their patient-provider experiences and its impact. We aimed to identify if there were similar themes and values across different disciplines and what we could learn about the participants beyond the 6 session prompt topics. In other words, we conducted an inductive analysis of the learners' narratives to discover what and how they were learning about being a clinician in their own words.

To systematize this process, authors 1 and 3 followed the phases of thematic analysis [32] by familiarizing ourselves with the narratives and conducting separate content analyses (coding) of all 54 stories. We analyzed for words and phrases found across all 6 sessions that were not the prompt topics (e.g., compassion). Using this ‘bottom up’ method allowed us to read the narratives “without trying to fit it into a preexisting coding frame, or the researcher's analytic preconceptions” [32, p. 83]. After initial coding, we searched for themes and then met to review our second-level analysis of the narratives to determine a rich description of the data set [32]. The third step involved discussing similarities, differences, and overlaps in the themes that were coded. We compared our interpretation of the codes that revealed four primary themes. The authors then defined the themes and returned to the participants' stories a final time to identify narratives that exemplified each theme.

The four themes that were identified were 1) feelings and emotions, 2) time management, 3) self- and other-awareness, and 4) work-life balance. The first theme was defined as specific emotional language that was used to demonstrate strong and overwhelming feelings. We identified words such as pain, joy, guilt, fear, suffering, stress, heartbreaking, uplifting, gratitude, helpless, frustrated, and empathy. A majority of the learners' narratives expressed the strong feelings they felt in relation to their clinical work. The authors coded for specific emotional terms to develop this category and recognized this as the most prevalent theme in the sessions. For example, the words “joy” and "pain" were both mentioned in four separate entries. In the following narrative, the participant uses multiple emotions to describe how they felt.

…*it was awesome to see the joy on their faces by me just spending time with them and allowing them to realize I was one of them. An African kid with the same elements of understanding they tried to convey to me. I felt pain and joy all at once because I knew the pain they were enduring.*

Awareness of one's emotions is a critical part of medical education [36], and we were intrigued by the learners' explicit statements of how they felt and ability to name the emotions that were experienced during their stories. In another example, the learner describes a patient with colorectal cancer and describes feeling “helpless” at the very end of the story (see Table 3). We occasionally came across stories filled with feeling, but the specific emotion was not always explicitly stated. In the following example, the learner does not name how they are feeling, nor do they identify a specific emotion. However, other learners recognized that an emotional response such as frustration or defeat was present in the narrator's story.

*There are worms in my stomach.*

*They are invisible worms.*

*They will kill me.*

*You can't help.*

*Everyday my patient repeats the same story.*

*Week after week.*

*The more I offer support.*

*It seems.*

*The stronger those worms get.*

**Table 3:** Sample Participant Stories by Theme.

Major themes	Sample stories
Feelings and emotions	In my 4th year of medical school on an inpatient internal medicine rotation one of my patients, a 40-year-old, father of 2 young children was diagnosed with terminal colorectal cancer. He had not come to accept his diagnosis – all he could say was ‘Why me? I can't believe this is happening to me. What about my wife, my kids – I am supposed to be their father, a husband.” I felt helpless. I had no response.
Time management	Despite being overwhelmed by my time constraints and work responsibilities as an intern, I took particular time to use a card with the alphabet and “yes/no” on it so she could point, and I would spend time talking to her about how she was doing and how I could help. She opened my eyes to struggles I couldn't have previously imagined.
Self-and other-awareness	…often when we think of “drug addicts” we envision particular individuals, for example who come to the hospital with questionable c/o [complaints] and seek drugs. There was a woman who came to the hospital. She did heroin. But, she had quite the story of death and suffering. Unfortunately, the label she had on her made others treat her differently. It made me angry. But I listened and helped her regardless.
Work-life balance	Weekend call. I don't want to be here. It's my cousin's wedding tonight and it's two hours away. I hope to get off by 2, 3 at the latest. But what about all my patients? They need quality care even though it's Saturday. And my consult…she just lost her mother and had a panic attack. And my other consult, she just had a baby but has psychosis. And the wedding, it's 2 h away and starts at 4:30, and I'm still at work at 4:30. This is what I signed up for.

The theme of time management was defined in relation to time constraints, restrictions, and pressures. This included participants feeling busy and often overwhelmed with work-related challenges. Frequently in the learners' stories, time was viewed as a hindrance to completing work as in the following example.

*I stay there in silence, trying to be respectful. The prayers keep going and going. I always thought I would be the type of person who would pray with a patient in need. But she kept going and going, and I was looking at the clock, when will she stop?*

In some circumstances, however, learners challenged their understanding of time in clinical contexts, choosing to spend more time with patients despite work-related pressures. An example of time with patients can be found in Table 3. In this story, the theme of patient-provider communication (PPC) also emerges since the learner reflects on their attempts to be respectful toward the patient.

A third theme of self- and other-awareness was discovered by the authors. This theme was defined as increased awareness of participants (e.g., healthcare role) and others (e.g., patients, coworkers). Sometimes the story exemplified how there was both an awareness of themselves as clinicians *and* awareness of the other person's perspective and how this impacted the patient-provider relationship, as was evident in the following story.

*Agitated, yelling, cursing, standing over me, five security[staff] behind me ready to protect [me]. Name calling, trying to scare me. I knew my role. Through anger and mood swings, I spent several days regularly seeing [patient], [I] showed compassion, and respect. Looking from others perspective, everyone has a reason and story behind their behaviors.*

In this example and in others (see Table 3), learners named their awareness of how the patient might have felt and how other clinicians were treating them. Because of their increased awareness, we found several examples where learners changed their behavior with patients, thereby demonstrating an ability to apply effective patient-provider communication. In the aforementioned example, we see the creation of a meaningful interaction because the learner is showing compassion and respect.

The final theme was work-life balance. This theme addressed the challenges learners faced trying to balance clinical care with their personal lives. For example, sometimes they questioned their clinical role and requirements (see Table 3) and other times learners identified how they coped with work-and life-expectations, as was evident in the following story.

*First year of residency was difficult especially with new experiences in my career and personal life. Here I was a new resident, wife, and mother of a newborn. I am truly grateful for my family who supported me through that difficult time. I don't think I would have made it through intern year without them*.

As graduate-level learners, participants were able to name the tensions they felt between clinical work and obligations or activities with friends and relatives. Although the stories did not always reveal if the tension was resolved, they did highlight how participants were learning to identify and address the challenges they were already facing as full-time healthcare professionals in training.

It is important to note that we often found multiple themes in the learners' narratives. As the authors read and re-read the participants' stories to identify examples for each theme, we recognized multiple themes across many of the narratives. For example, in the following story, the learner addresses their feelings and an awareness of the patient in relation to themselves and their family.

*Covid patients in ICU. Psych consults, know prognosis is bleak, realize that they must reach out to family to rebuild relationships, connect with loved ones because they realized any day may be their last. Patients consistently cited family as largest support, their motivation and source of joy. From a family of 3 brothers and 3 sisters, with 35 cousins I'm close with, I felt that. Family matters most.*

Another example of multiple themes involves a participant's story about how they feel writing a narrative. The story addresses the learner's feelings and emotions, self-awareness, and struggles with work-life balance.

*I'm feeling blocked. I have examples, cases of IDM, even from today. The truth is I don't have the energy to write a story. It is frustrating at times dealing with other people's expectations of what your job is/should be. Hopefully tomorrow will be better.*

There were many other examples where learners addressed multiple themes in a single story. To clarify, the themes that we coded were in addition to the session topics (e.g., compassion), revealing how learners were making sense of their role as healthcare providers in their own words.

## 4 Discussion and conclusion

### 4.1 Discussion

This longitudinal, interdisciplinary narrative project demonstrates how learners were able to personally reflect, collaboratively share, and later apply what they learned, almost immediately. Through reflection and writing in a safe environment, participants practiced patient-provider communication (PPC) skills and learned about resiliency and wellness. These results were demonstrated consistently across the entire project.

Quantitative analysis shows the narrative project had a positive impact on wellness, resilience, and listening skills, and that most learners felt they could use this narrative tool in their personal or professional lives with patients and team members. One of the limitations of our analysis is that we used descriptive statistics due to the small sample size. Therefore, we were unable to conduct inferential comparisons because of the increased risk of type 2 error. Qualitative analysis of the open-ended survey questions revealed themes of wellness, listening, and patient-centered care. A thematic analysis of the learners' 54 stories revealed new themes of feelings and emotions, time, self- and other-awareness, and work-life balance. Due to the small number of participants from each program , we recognize the limitations of a small sample size in our research. However, the narratives we collected revealed a rich description of the data set and testimony to the impact of the narrative project on the growth and development of graduate learners.

The Write-Read-Reflect (WRR) process focuses on learners and their reflection of clinician practices with peers as a group. Unlike other models of medical education that teach interprofessionalism in a one-day workshop about interactions during rounds, the structured WRR process allows learners to gain insight personally and professionally by being present to others' stories and recognizing the value of having team members from different learning levels and specialties. This process is learner focused and primarily learner driven. Although a facilitator assisted in the reflection process, we noticed that learners felt more comfortable and confident to co-facilitate discussion. By the third and fourth sessions, learners were asking each other reflective questions and participating in facilitator responsibilities. The WRR process encourages learner-led reflection because of the emphasis on sharing one's story and learning to listen and respond to others' stories. What stood out in the results were participants who recognized another team member's expertise and demonstrated an appreciation of their role during clinical encounters. This educational innovation, therefore, fills a gap for how learning about effective communication and teamwork, over time, can occur with other team members. And, this approach helped learners recognize similar challenges that caregivers from other departments might face when working with patients. Ultimately this process can help team cohesiveness and build trust among team members.

In review of the individual narrative session surveys, there was a significantly different response from the 13 who completed the end of project survey compared to the 69 responses from the individual narrative sessions. Learners who thought participation in the project was not helpful were primarily from the HPM fellows. There was also some ambivalence demonstrated by the pastoral care (PC) residents as well about the overall value of participating in the narrative project. Programs that are short (e.g., HPM) or with overlap of curriculum content (e.g., PC) enjoyed and benefited from exposure to the WRR narrative process although they may also benefit from fewer sessions. Programs that are longer or have less curriculum overlap (e.g., FM, PSY) reported value in the monthly sessions and in their overall narrative project experience. We recognize there are fundamental curriculum differences and experiences between the FM and PSY residents compared to the PC residents and HPM fellows. There may also be a benefit to keeping the HPM fellows and PC residents together due to considerable overlap in patient population.

### 4.2 Innovation

We developed an interdisciplinary narrative program derived from narrative medicine [33] and developed from a clinical perspective [29]. This type of narrative inquiry was applied to expand the GME curriculum. The goal was to develop an interprogram, interlevel, interdisciplinary narrative curriculum to advance learner wellness and improve patient-provider communication skills. Program directors from pastoral care (PC), hospice and palliative medicine (HPM), psychiatry (PSY), and family medicine (FM) recognized the potential educational benefits of a longitudinal narrative program because of the use of reflective practice to teach common curriculum topics such as compassion, stress, interdisciplinary teamwork, imminently dying, ethics, and religion/spirituality. Based on two years of positive feedback from GME learners, program directors and a national recognition of educational value, the program

has been implemented in the fourth-year curriculum of the medical school and continues to be expanded among the other GME programs. The narrative program has become a standard part of the four disciplines' curricula. Key to this expansion was employing a part-time, narrative medicine trained facilitator.

Narrative education programs are a powerful innovation that fosters transformational and interprofessional learning, impacts patient experience, prevents burnout, and reconnects healthcare professionals to their core sense of meaning and purpose, all of which benefits caregivers, health systems, and the larger community. First, narrative education utilizes healthcare workers' stories in clinical practice and education as a way to promote coherence, purpose, and existential mattering. Narrative exchange models [29] are one such technique to support our healthcare workers' sense of meaning and purpose. Second, from the perspective of the fourth author, an ACGME Designated Institutional Officer (DIO), narrative medicine offers sponsoring institutions of post-graduate education a favorable cost/benefit analysis and readiness for practice strategy, especially as more health systems across the country are starting resident and fellow programs to grow their physician workforce [37]. Physicians remain at a 40% higher risk of occupational burnout than workers in other fields [34]. Concurrently, it has been estimated that the cost of physician burnout to an organization can range from $250,000 to more than $1 million per practicing clinician. These costs include recruitment and replacement, loss of billables, medical errors, higher malpractice risk, and reduced patient satisfaction [35]. Setting up a narrative curriculum in a residency consists mostly of facilitator training, administrative coordination expenses, and time, a fraction of the cost of burnout once in independent practice. Implementing narrative medicine curriculum into a residency program gives residents the skills to curb their future risk of burnout. Given this cost/benefit analysis, DIOs and other GME leaders should encourage pilots and longitudinal evaluation of this education innovation as an area for future research.

### 4.3 Conclusion

We recommend the narrative project be tailored to the needs of each individual GME program. For example, prompts can be changed according to curricula needs (e.g., ACGME milestones).

Programs that are shorter in length (e.g., HPM) or those with significant overlap of curriculum where topics covered in the prompts are in the existing curriculum (e.g., PC) seem to enjoy and benefit from exposure to the WRR narrative tool although they may also benefit from fewer sessions. Other programs that are longer (e.g., 3+ years) and with less overlap of curriculum (e.g., FM, PSY) reported value in the monthly sessions and in their overall narrative project experience.

Developing the series and orchestrating the many, moving parts required for an interdisciplinary educational program was a challenge at times. We needed buy-in from the program directors to suggest the integration of new curriculum into each program. This was a significant request and required meetings and early demonstrations of the WRR narrative process. There were scheduling challenges that are common with interdisciplinary education [3]. Faculty represented different disciplines which required coordination across multiple programs and schedules. For the interdisciplinary aspect of this process to work, program directors and coordinators needed to periodically meet and ensure everyone was on the same page.

After running the narrative project for a few years, we recognize there is a needed change to the facilitators. If programs cannot train facilitators in the WRR process, an alternative option is to have a trained educator with a background in humanities or behavioral sciences combined with the ability to facilitate learner-led reflective discussions. Training is recommended for session facilitators who desire additional education in emotional intelligence to be comfortable leading such reflective discussions on emotionally charged topics (e.g., working with imminently dying patients). With a large group of learners, the small breakouts are solely learner-led and encourages applying effective patient-provider communication and listening skills in a safe environment. The facilitator's role is to oversee group discussion and keep learners on track with the session.

In debriefing meetings with program directors and faculty, we were amazed by the response of how often their learners had an “A-ha” moment. One program director commented, “They [learners from other specialties] didn't know what we did. Now they do.” Other faculty commented on how learners' small epiphanies during the narrative sessions later turned into larger realizations about the value of other' perspectives in their clinical workspace. Learners felt this made them better team members and helped them place more of the patient's chosen words into the clinical record. In short, learners recognized that interdisciplinary teamwork and patient-provider communication (PPC) matters, and they learned how to be more effective in team member communication. This ultimately effected their overall communication by focusing on the words being spoken. Healthcare providers continue to tell us that sharing their stories using our facilitated narrative method leaves them feeling validated and supported. These narrative experiences allow them to practice critical listening skills they can use immediately with colleagues and patients as well as leaving with an enhanced feeling of professional resilience.

## Ethics statement

This study was conducted with prior approval from the Institutional Review Board at St. Luke's University Health Network.

## Statement participant identifiers

We confirm that all personal identifiers have been removed or disguised, so that the participants are not identifiable and cannot be identified through the details of this manuscript.

## Funding

The narrative project was supported by St. Luke's University Health Network. This research did not receive any grants from funding agencies in the public, commercial, or not-for-profit sectors.

## Declaration of Competing Interest

The authors declare the following financial interests/personal relationships which may be considered as potential competing interests.

Lorraine Dickey and Vivian Foulke reports a relationship with The Narrative Initiative that includes: employment.
